# Factors influencing nutritional literacy among rural older adults: a cross-sectional survey based on the theory of planned behavior

**DOI:** 10.3389/fnut.2025.1578836

**Published:** 2025-07-07

**Authors:** Shasha Liu, Xiaomei Fan, Li Jiang, Tao Liu

**Affiliations:** ^1^Anesthesia and Surgery Center of Chengdu BOE Hospital, Chengdu BOE Hospital, Chengdu, China; ^2^First Affiliated Hospital of Chengdu Medical College, Chengdu, China; ^3^Department of Nursing, Jinzhou Medical University, Jinzhou, China

**Keywords:** rural older adults, nutritional literacy, nutritional attitudes, social support, activities of daily living, mediating role

## Abstract

**Objective:**

To investigate the factors influencing nutritional literacy among rural elderly individuals in northern Sichuan based on the theory of planned behavior (TPB).

**Methods:**

A convenience sampling method was employed to recruit 311 elderly participants (aged ≥60 years) from Sanlin Village (Cangxi County, Guangyuan City), Puji Village (Yilong County, Nanchong City), and Luhua Village (Bazhou District, Bazhong City). The survey instruments included general demographic questionnaires, the nutrition attitude scale (NAS), the multidimensional scale of perceived social support (MSPSS), the instrumental activities of daily living scale (IADLS), the general self-efficacy scale (GSES), and the nutrition literacy questionnaire for the Chinese elderly (NLQ-E). Data were analyzed using SPSS 26.0 and AMOS 23.0.

**Results:**

Hierarchical regression analysis revealed that education level positively predicted nutritional literacy (β = 0.126, *t* = 2.659, *P* < 0.01), while denture use due to tooth damage negatively predicted nutritional literacy (β = −0.077, *t* = −2.375, *P* < 0.05). Nutrition attitude (β = 0.312, *t* = 6.662, *P* < 0.001) and perceived social support (β = 0.243, *t* = 4.283, *P* < 0.001) were positive predictors, whereas daily living ability exhibited a negative predictive effect (β = −0.251, *t* = −4.445, *P* < 0.001). Structural equation modeling (SEM) indicated that nutrition attitude had a direct effect of 0.333 and an indirect effect of 0.050 on nutritional literacy. Daily living ability showed a direct effect of −0.481 and an indirect effect of −0.101. Self-efficacy partially mediated the relationships between nutrition attitude and nutritional literacy, as well as between daily living ability and nutritional literacy.

**Conclusion:**

Elderly individuals in northern Sichuan with more positive nutrition attitudes, higher perceived social support, and stronger daily living abilities demonstrated higher nutritional literacy. Self-efficacy partially mediated the influence of nutrition attitude and daily living ability on nutritional literacy.

## 1 Introduction

China has witnessed a continuous increase in the proportion of older adults since entering an aging society in 1999. According to the 2023 National Economic and Social Development Statistical Bulletin of the People's Republic of China, by the end of 2023, individuals aged 60 and above accounted for 21.1% of the total population (1). A survey revealed that 75.8% of older adults aged 60+ suffer from at least one chronic disease (2). This substantial aging population base imposes escalating caregiving and economic burdens on numerous families, while simultaneously posing intensified challenges to the healthcare system (3). Therefore, promoting healthy aging has become a critical strategy for families and society to address these challenges.

Nutritional status serves as a critical foundation for preventing chronic diseases and maintaining health in the elderly population (4). The prerequisite for determining the nutritional status of older adults lies in their behavioral capacity to promote nutrition. The behavioral capacity of individuals to understand nutritional knowledge and guide their own nutrient intake is formally defined as nutritional literacy (5). The concept of nutritional literacy originates from health literacy and constitutes an essential component of health literacy (6–8). Therefore, enhancing the nutritional literacy level of older adults holds significant importance for promoting their overall health.

The key to improving the nutritional literacy of the elderly lies in identifying its influencing factors and implementing targeted interventions based on these factors. Existing research has explored some determinants of nutritional literacy. Japanese scholar Aihara and Minai found that elderly women in local communities exhibited lower nutritional literacy due to their limited educational attainment and socioeconomic status (9), confirming correlations between nutritional literacy and factors such as gender and education level among the elderly. Similarly, a large-scale survey in China revealed that older adults in western rural areas with lower educational levels demonstrated constrained nutritional literacy due to insufficient dietary knowledge (10). A Dutch study demonstrated that selecting nutritious yet affordable foods by socioeconomically disadvantaged populations could help narrow disparities in nutritional literacy (11). Additionally, oral/dental health, self-care capacity, and family structure have also been identified as significant influencing factors for nutritional literacy in the elderly (12–14). However, current investigations indicate a lack of systematic analysis regarding the comprehensive influencing factors of nutritional literacy in aging populations (15).

The theory of planned behavior (TPB), evolved from the multi-attribute attitude theory, was refined and published by Ajzen in 1991 (16). Its core elements consist of behavioral attitudes, subjective norms, and perceived behavioral control, which collectively shape an individual's behavioral intention, directly determining their actual behavior (17). An individual's behavioral beliefs form the cognitive and affective foundation for these three variables, while personal and sociocultural factors also significantly influence behavioral intentions (18). However, in daily life, identical behavioral intentions do not always lead to consistent behavioral outcomes. Consequently, scholars have proposed the existence of mediating variables between behavioral intention and actual behavior. These mediators, representing an individual's capacity to execute actions, are termed implementation intentions (19). Currently, the theory of planned behavior has been validated as a theoretical framework for explaining food acquisition-related behaviors, systematically elucidating the evolution of such behaviors (20).

The Sichuan Basin is characterized by a dietary culture marked by heavy use of salt and oil, particularly favoring preserved foods such as cured meats, sausages, pickled vegetables, and paocai (fermented vegetables), habits that have posed significant health risks to local populations (21). In northern Sichuan, predominantly mountainous and historically economically underdeveloped, rural areas have seen substantial improvements in infrastructure, transportation, healthcare, social welfare, and income levels following the targeted poverty alleviation campaign (22). However, a notable gap persists in personal health literacy between rural residents and urban populations (23). While disparities in nutritional literacy stemming from dietary habits are influenced by group attributes, individual cognition remains the dominant driver of such behaviors. In the context of investigating factors affecting nutritional literacy among rural older adults, the theory of planned behavior (TPB) provides a robust framework to explain how individual perceptions and self-efficacy collectively shape dietary behaviors. Moreover, the actionable nature of the TPB model is critical for addressing the pervasive “knowledge-action gap” observed in rural aging populations compared to their urban counterparts. To assess the nutritional behavior capacity of elderly individuals in this region, this study employs the theory of planned behavior (TPB) to systematically analyze factors influencing nutritional literacy, aiming to provide a theoretical foundation for advancing healthy aging initiatives in the area.

## 2 Theoretical background

**Figure 1:**
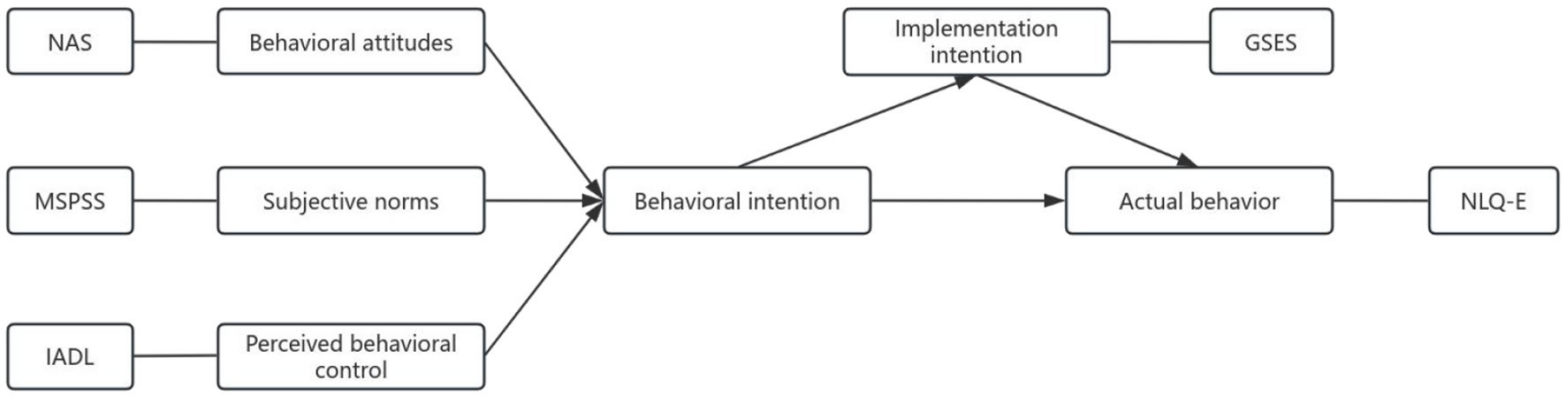
TPB model path.

The TPB posits that behavioral intention (shaped by attitudes, subjective norms, and perceived behavioral control) drives action, with self-efficacy acting as a mediator between intention and behavior. In our model, nutrition attitude reflects behavioral beliefs, perceived social support aligns with subjective norms, and daily living ability (IADL) operationalizes perceived control. Self-efficacy bridges intention to actual behavior. Based on the elements of the theory of planned behavior (TPB), the following variables were selected to construct the theoretical framework of this study and to examine the influencing factors of nutritional literacy (Figure 1).

## 3 Methodology

### 3.1 Study participants

This study employed a convenience sampling method to select elderly participants aged ≥60 years from Sanlin Village (Cangxi County, Guangyuan City, Sichuan Province), Puji Village (Yilong County, Nanchong City), and Luhua Village (Bazhou District, Bazhong City) between June 2023 and October 2023. Inclusion criteria required participants to have resided in local rural areas for ≥6 months, possess clear consciousness, exhibit no language comprehension or communication barriers, and voluntarily participate in the study. Exclusion criteria included individuals diagnosed with Alzheimer's disease or other mental disorders (with formal medical diagnostic reports), those refusing to cooperate with the investigation, and individuals completely dependent on others for daily living. The sample size was determined as 5–10 times the number of variables, ultimately enrolling 311 rural elderly participants (questionnaire recovery rate: 92%). This study was approved by the Ethics Committee of Jinzhou Medical University (Approval No.: JZMULL2022108).

### 3.2 Data collection

Three local community workers with extensive familiarity with the elderly population were selected as investigators for this study. The three investigators underwent uniform training to ensure comprehension of the research objectives and methodologies, as well as proficiency in appropriately utilizing survey tools for data collection. Core members of the research team subsequently evaluated the investigators, and upon passing the assessment, they conducted a pilot survey followed by the formal survey. To enhance their motivation, a compensation of five RMB per completed questionnaire was provided.

### 3.3 Measures

#### 3.3.1 General demographic data

This section was self-designed by the researchers based on a review of domestic and international literature and aligned with the objectives of the study. It included: age, gender, marital status, educational level, personal monthly living expenses, dental status, number of chronic diseases, and self-rated health status.

#### 3.3.2 Nutrition attitude scale (NAS)

The Nutrition Attitude Scale (NAS) used in this study was developed by Chinese scholar Jiang Ting in 2021 to investigate the correlation between older adults' nutritional attitudes, knowledge, and compliance with dietary guidelines (24). It consists of one dimension and six items. For this survey, researchers applied a Likert 5-point scoring system to the six items based on the study's objectives: “strongly disagree” = 1 point, “disagree” = 2 points, “neutral” = 3 points, “agree” = 4 points, and “strongly agree” = 5 points. The total score ranged from 6 to 30, with higher scores indicating more positive rational nutritional attitudes among older adults. The Cronbach's α coefficient of this scale in the current study was 0.897.

#### 3.3.3 Multidimensional scale of perceived social support (MSPSS)

There are numerous existing versions of social support scales (25). Based on the theoretical path analysis of this study, the multidimensional scale of perceived social support (MSPSS) developed by Zimet et al. (26) was selected for its high alignment with the purpose of assessing individuals' perceived social support. Chinese scholar Huang Li (27) translated and adapted the scale into Chinese in 1996. The scale demonstrates a Cronbach's α coefficient of 0.817 and has shown good reliability and validity across various population studies. The scale comprises 12 items across three dimensions: family support (four items), friend support (four items), and significant other support (four items). It employs a Likert 7-point scoring system: 1 = “strongly disagree,” 2 = “disagree,” 3 = “slightly disagree,” 4 = “neutral,” 5 = “slightly agree,” 6 = “agree,” and 7 = “strongly agree.” Total scores range from 12 to 84 points, with higher scores indicating greater perceived social support levels.

#### 3.3.4 Instrumental activities of daily living scale (IADL)

Developed by American scholars Lawton and Brody to assess an individual's ability to perform essential activities for independent living (28), the IADL scale includes eight items: telephone use, housekeeping, laundry, shopping, meal preparation, medication management, transportation use, and financial management. The total score reflects the individual's current functional status, with a minimum score of 8 indicating full independence and scores above 8 (up to a maximum of 31) indicating varying degrees of functional decline. Each item is scored from 1 (normal function) to 4 (significant impairment), with scores ≥3 in two or more items or a total score ≥22 indicating marked functional disability. The scale demonstrates a Cronbach's α coefficient of 0.883.

#### 3.3.5 General self-efficacy scale (GSES)

The general self-efficacy scale, revised by Schwarzer (1995), quantifies individuals' psychological resilience and confidence when facing setbacks or challenges. The original version contained 20 items, which were later refined in 1997 into a 10-item single-dimension scale. The total self-efficacy score is calculated by summing the scores of all 10 items. Responses are rated on a Likert 4-point scale: 1 = “completely incorrect,” 2 = “somewhat correct,” 3 = “mostly correct,” and 4 = “completely correct.” This scale has been translated into 25 languages and is widely used globally. The Chinese version of the GSES employed in this study demonstrates a Cronbach's α coefficient of 0.890 (29).

#### 3.3.6 Nutrition literacy questionnaire for the Chinese elderly (NLQ-E)

The NLQ-E, developed by Professor Zhang from the School of Public Health at Peking University in 2022 (30), is a 20-item core questionnaire comprising three dimensions: items 1–6 assess elderly individuals' understanding of nutritional knowledge, items 7–15 evaluate their awareness of healthy lifestyles and dietary behaviors, and items 16–20 measure their proficiency in nutrition-related skills. Responses are rated on a Likert 5-point scale: 1 = “strongly disagree,” 2 = “disagree,” 3 = “neutral,” 4 = “agree,” and 5 = “strongly agree,” yielding a total score ranging from 20 to 100, with higher scores indicating better nutrition literacy. In this study, the scale demonstrated a Cronbach's α coefficient of 0.931.

### 3.4 Data analysis

This study employed SPSS26.0 software for statistical analysis. First, descriptive statistical analyses were applied to general demographic data and scale measurements: categorical data were expressed as frequencies and percentages, while continuous data were presented as mean ± standard deviation. Second, independent samples *t*-tests and ANOVA were used to examine differences in nutritional literacy based on general demographic characteristics. Pearson correlation analysis was adopted to explore relationships between nutritional literacy and nutritional attitudes, social support, daily living abilities, and self-efficacy. Hierarchical regression analysis was further utilized to identify influencing factors of nutritional literacy. Finally, a structural equation model (SEM) was constructed using AMOS 26.0 software, with the Bootstrap method applied to verify the mediating effect of self-efficacy between behavioral intentions (formed by nutritional attitudes, social support, and daily living abilities) and nutritional literacy (representing actual behaviors of the elderly). The significance level was set at α = 0.05.

## 4 Results

### 4.1 Univariate analysis of nutritional literacy in the elderly based on demographic variables

A total of 336 questionnaires were distributed in this study, with 311 valid questionnaires collected, yielding a response rate of 92.5%. The results revealed statistically significant differences in nutritional literacy scores among elderly individuals based on age, gender, marital status, educational level, monthly personal living expenses, dental status, number of chronic diseases, and self-rated health status (*P* < 0.05). Details are presented in Table 1.

**Table 1:** Univariate analysis of nutritional literacy in the elderly based on demographic variables (*n* = 311).

**Variable**	**Category**	***n***	**Percentage (%)**	**Nutritional literacy score**	***F*/*t* value**	***P* value**
Age	60–69 years	144	46.3	84.28 ± 11.63	62.93	<0.001
	70–79 years	122	39.2	73.83 ± 15.99		
	≥80 years	45	14.5	57.33 ± 17.62		
Gender	Male	154	49.5	82.27 ± 15.15	6.53	<0.001
	Female	157	50.5	70.41 ± 16.80		
Marital status	Unmarried	1	0.3	79.00	8.09	<0.001
	Unmarried	230	74.0	78.52 ± 16.33		
	Divorced/Widowed	80	25.7	69.83 ± 17.60		
Educational level	Illiterate	117	37.6	63.50 ± 15.59	64.39	<0.001
	Primary school	111	35.7	80.09 ± 12.79		
	Middle school	80	25.7	88.96 ± 10.67		
	High school or above	3	1.0	96.00 ± 6.93		
Monthly living expenses	<500 RMB	119	38.3	65.23 ± 16.62	48.96	<0.001
	500–999 RMB	133	42.8	79.56 ± 13.05		
	1,000–1,999 RMB	53	17.0	91.43 ± 9.61		
	≥2,000 RMB	6	1.9	89.17 ± 14.11		
Dental status	Intact teeth	82	26.4	86.77 ± 10.71	17.82	<0.001
	Damaged teeth with dentures	117	37.6	74.07 ± 18.82		
	Damaged teeth without dentures	109	35.0	71.49 ± 15.73		
	Edentulous	3	1.0	57.00 ± 14.93		
Number of chronic diseases	0	141	45.3	84.65 ± 11.81	30.13	<0.001
	1	128	41.2	70.91 ± 17.14		
	2	40	12.9	65.75 ± 18.07		
	≥3	2	0.6	41.00 ± 1.41		
Self-rated health status	Poor	44	14.1	59.91 ± 19.98	68.46	<0.001
	Fair	152	48.9	72.69 ± 14.42		
	Good	115	37.0	87.30 ± 10.99		

### 4.2 Correlation analysis of nutritional literacy, nutritional attitudes, perceived social support, daily living abilities, and self-efficacy in the elderly

In this study, Pearson correlation analysis was employed. The results demonstrated that nutritional literacy and its subdimensions in the elderly were significantly positively correlated with nutritional attitudes, perceived social support, daily living abilities, and self-efficacy (*P* < 0.01), with specific correlation coefficients detailed in Table 2.

**Table 2:** Correlation analysis of nutritional literacy and nutritional attitudes in the elderly (*n* = 311).

**Item**	**NLQ-E**	**Knowledge**	**Behavior**	**Skills**
NAS	0.769^**^	0.780^**^	0.740^**^	0.742^**^
MSPSS	0.794^**^	0.768^**^	0.774^**^	0.780^**^
IADL	−0.770^**^	−0.771^**^	−0.774^**^	−0.764^**^
GSES	0.784^**^	0.785^**^	0.752^**^	0.775^**^

** *P* < 0.01.

### 4.3 Hierarchical regression analysis of influencing factors on nutritional literacy in the elderly

To validate the influencing factors of nutritional literacy in the elderly, this study employed hierarchical regression analysis based on the theory of planned behavior (TPB) model. Nutritional attitudes, perceived social support, daily living abilities, and demographic variables showing statistical significance in the univariate analysis were included as independent variables, with nutritional literacy as the dependent variable. Prior to regression analysis, nominal categorical variables were standardized through dummy coding. Multicollinearity diagnostics revealed that marital status exhibited multicollinearity and was subsequently excluded from the model. The variance inflation factor for all remaining predictors was <5, confirming their inclusion in the final model. The results indicated that educational level, dental status, nutritional attitudes, perceived social support, and daily living abilities collectively explained 75.0% of the variance in nutritional literacy (*F* = 78.650, *P* < 0.001). The coding methods for independent variables are presented in Table 3, and regression results are detailed in Table 4.

**Table 3:** Assignment and dummy coding of independent variables.

**Variable**	**Coding method**
Age	60–69 years = 1; 70–79 years = 2; ≥80 years = 3
Gender	Male = 1; Female = 2
Educational level	Illiterate = 1; Primary school = 2; Middle school = 3; High school or above = 4
Monthly living expenses	<500 RMB = 1; 500–999 RMB = 2; 1,000–1,999 RMB = 3; ≥2,000 RMB = 4
Dental status	Intact teeth (reference) = 1, 0, 0, 0; Damaged teeth with dentures = 0, 1, 0, 0; Damaged teeth without dentures = 0, 0, 1, 0; Edentulous = 0, 0, 0, 1
Number of chronic diseases	0 = 1; 1 = 2; 2 = 3; ≥3 = 4
Self-rated health status	Poor = 1; Fair = 2; Good = 3

**Table 4:** Hierarchical regression analysis of factors influencing nutrition literacy.

**Variable**	**Level 1**		**Level 2**		**Level 3**		**Level 4**	
	β	***t***	β	***t***	β	***t***	β	***t***
Age	−0.219	−4.508^***^	−0.070	−1.673	−0.045	−1.141	0.020	0.495
Gender	−0.041	−0.809	−0.022	−0.520	0.030	0.773	0.006	0.161
Educational level	0.287	4.685^***^	0.188	3.680^***^	0.170	3.563^***^	0.126	2.659^**^
Monthly living expenses	0.179	3.485^**^	0.077	1.780	0.021	0.513	0.037	0.939
Number of chronic diseases	−0.044	−0.683	−0.056	−1.048	−0.079	−1.589	−0.044	−0.904
Self-rated health status	0.249	3.675^***^	0.125	2.207^*^	0.064	1.191	0.035	0.663
Damaged teeth with dentures	−0.059	−1.360	−0.092	−2.544^*^	−0.078	−2.323^*^	−0.077	−2.357^*^
Damaged teeth without dentures	−0.027	−0.535	0.004	0.088	0.006	0.168	0.006	0.169
Edentulous	−0.007	−0.166	−0.024	−0.750	−0.011	−0.373	0.001	0.033
NAS			0.512	12.047^***^	0.324	6.726^***^	0.312	6.662^***^
MSPSS					0.357	6.856^***^	0.243	4.283^***^
IADL							−0.251	−4.445^***^
*R*^2^ after adjustment	0.547		0.694		0.735		0.750	
Δ*R*^2^	0.561		0.143		0.040		0.016	
*F*	42.676		71.314		79.045		78.650	
*P*	<0.001		<0.001		<0.001		<0.001	

* *p* < 0.05;

** *p* < 0.01;

*** *p* < 0.001.

### 4.4 Mediation effect analysis of influencing factors on nutritional literacy in older adults and establishment of structural equation model

**Figure 2:**
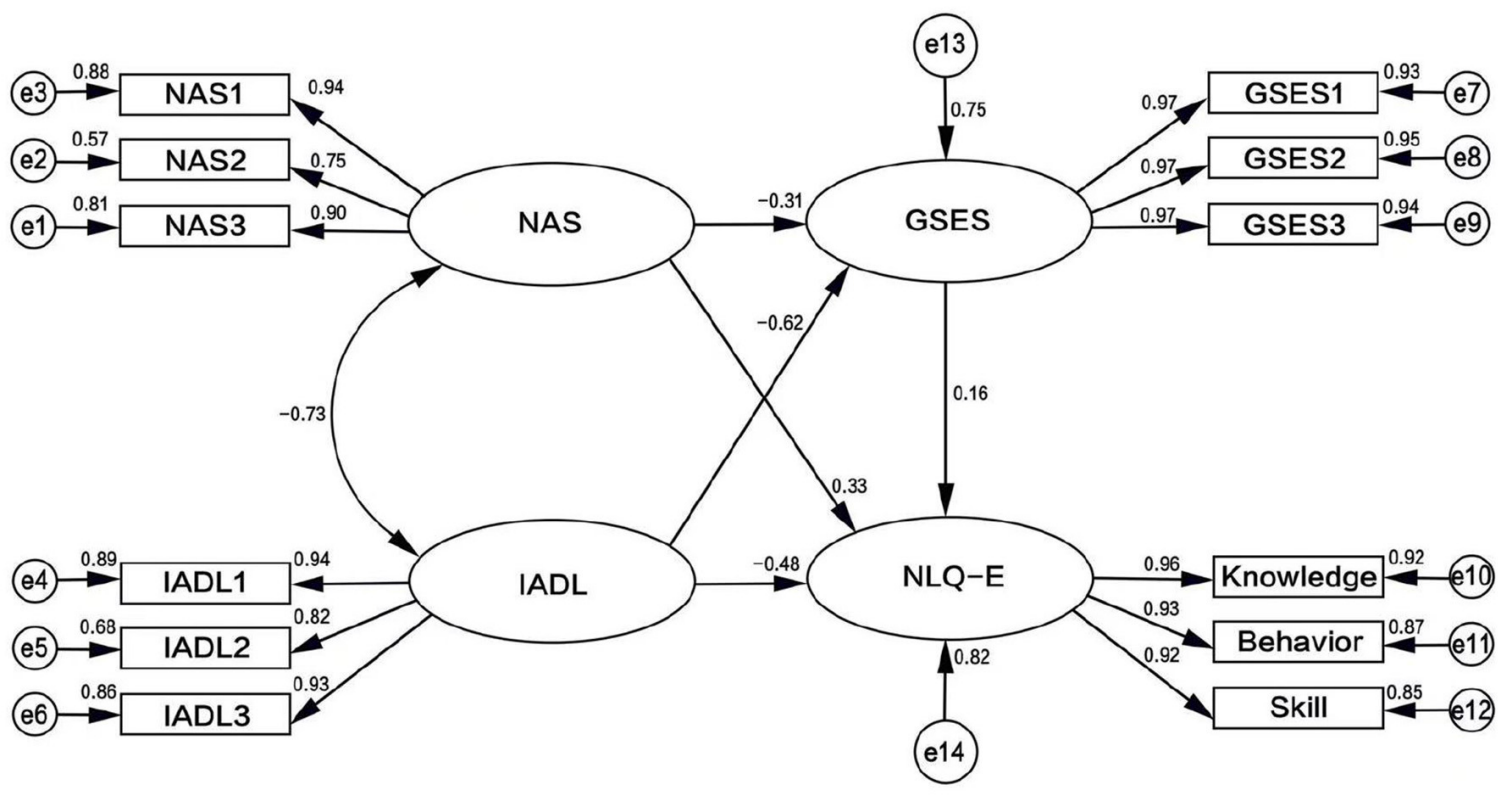
Structural equation model of nutritional attitude, activities of daily living, self-efficacy, and nutritional literacy in older adults.

Based on the theory of planned behavior (TPB), this study used the self-efficacy score of older adults (representing implementation intention) as a mediating variable to examine its effect between behavioral intention and actual behavior. The analysis was conducted using AMOS 26.0 to construct a structural equation model (SEM). Nutritional attitude and activities of daily living (ADL) were included as independent variable measurement models, self-efficacy as the mediating variable measurement model, and nutritional literacy as the dependent variable measurement model. Standardized path coefficients and the structural equation model are shown in Figure 2. Maximum likelihood estimation was employed for parameter evaluation, and the fitted model incorporating nutritional attitude, ADL, self-efficacy, and nutritional literacy demonstrated satisfactory fit indices meeting adaptation criteria (Table 5).

**Table 5:** Model fit data and fit indices adaptation criteria.

**Index**	**Acceptance criteria**	**Fit indices**
CMIN/DF	<3	2.964
GFI	>0.9	0.930
NFI	>0.9	0.971
RFI	>0.9	0.961
IFI	>0.9	0.981
TLI	>0.9	0.974
CFI	>0.9	0.981
RMSEA	<0.05 (Excellent), <0.08 (Acceptable)	0.080
SRMR	<0.05	0.041

During initial modeling, perceived social support was included in the independent variable measurement model, but the model fit failed to meet standards. Additionally, no existing domestic or international studies have incorporated perceived social support as an independent variable with self-efficacy as a mediating variable. Consequently, perceived social support was excluded from the final independent variable measurement model in this study.

Finally, this study employed the non-parametric percentile bootstrap method to calculate the mediating effect size of self-efficacy, with results presented in Table 6.

**Table 6:** Mediation effect analysis of self-efficacy (standardized).

**Effect type**	**Path relationship**	**Effect size**	**Boot SE**	***P-*value**	**95% Confidence interval**	
					**Lower**	**Limit**
Total effect	X1 → Y	0.383	0.068	0.001	0.258	0.524
Direct effect	X1 → Y	0.333	0.072	0.001	0.199	0.483
Indirect effect	X1 → Y	0.050	0.023	0.001	0.025	0.099
Total effect	X2 → Y	−0.582	0.062	0.001	−0.698	−0.457
Direct effect	X2 → Y	−0.481	0.066	0.001	−0.357	−0.626
Indirect effect	X2 → Y	−0.101	0.043	0.001	−0.020	−0.189

X1: nutritional attitude; X2: activities of daily living; Y: nutritional literacy. Bootstrap resampling was performed with 2,000 iterations.

Figure 2 shows standardized path coefficients. Nutritional attitude had the strongest direct effect on nutritional literacy (β = 0.333), while daily living abilities exerted a negative direct effect (β = −0.481). Self-efficacy partially mediated both relationships, with indirect effects of 0.050 (attitude → efficacy → literacy) and −0.101 (IADL → efficacy → literacy). Model fit indices (CFI = 0.981, RMSEA = 0.080) met acceptable thresholds.

## 5 Discussion

### 5.1 Multiple linear regression analysis of influencing factors of nutritional literacy in the elderly based on the TPB model

#### 5.1.1 Attitudes and nutritional literacy

Nutritional attitude reflects an individual's proactive approach toward nutrition-related issues (31, 32). In this study, higher nutritional attitude scores among elderly participants were associated with better nutritional literacy, indicating that a more positive engagement with nutritional challenges enhances their capacity to obtain adequate nutrition. A UK-based study investigating determinants of dietary behaviors among older adults similarly demonstrated that individuals with more health-conscious attitudes exhibited stronger associations with improved nutritional health scores (33). Furthermore, those who prioritize health considerations in food selection maintained higher dietary quality, whereas perceiving nutritional value as a barrier was linked to diminished dietary outcomes (34). Therefore, improving older adults' interest in nutrition acquisition—through measures such as nutrition knowledge campaigns, health lectures, and nutrition promotion activities—could enhance their motivation to pursue nutritional adequacy and health improvement, thereby advancing healthy aging. Although current research on the relationship between nutritional literacy and nutritional attitude remains limited, some studies using different assessment tools have confirmed their positive correlation (35, 36). However, the intrinsic connections among these three dimensions (attitude, literacy, and behavior) have not been thoroughly explored. This study therefore employs the theory of planned behavior (TPB) to analyze the relationship between attitudes and behaviors.

#### 5.1.2 Perceived social support as subjective norm

Perceived social support refers to an individual's assessment of the level of external support they receive when making decisions, including family support, friend support, and other forms of assistance. Providing effective social support can reduce social isolation and loneliness among the elderly (37), and the perceived degree of support can also change their behavioral processes and outcomes. For instance, regular family meals can boost the confidence of the elderly in their diet, thereby improving their nutritional status, etc. This study employed Pearson correlation analysis to examine the relationship between perceived social support and nutritional literacy. The results revealed a significant positive correlation, indicating that older adults with higher perceived social support tend to have higher nutritional literacy scores. Existing studies have confirmed the positive impact of social support on older adults' literacy and even their proactive engagement in acquiring nutrition (38, 39). Therefore, to enhance nutritional literacy among older adults, targeted measures should be implemented based on their diverse needs, including support from families, relatives, friends, and broader societal initiatives to ensure sustained attention and assistance for this population.

#### 5.1.3 IADL as perceived behavioral control

The findings of this study reveal a significant correlation between older adults' daily living abilities and their nutritional literacy. The Instrumental Activities of Daily Living (IADL) scale evaluates functional impairment based on older adults' ability to use tools in daily life. In rural areas, older adults primarily rely on self-cooking and purchasing to obtain nutrition (40), thus those with stronger daily living abilities tend to exhibit higher nutritional literacy. Currently, direct research on the relationship between nutritional literacy and daily living abilities remains limited. However, studies have reported that older adults' daily living abilities directly influence their quality of life and health status (41, 42). As nutritional literacy serves as a foundational behavioral competency for maintaining health, this indirectly supports the observed association. Therefore, governments and society should prioritize improving rural infrastructure and facilities, such as convenience stores, drinking water systems, public transportation services, healthcare centers, and senior activity centers. In summary, providing accessible living facilities to mitigate disability caused by complications from chronic diseases can substantially enhance older adults' daily living capacities, thereby positively elevating their nutritional literacy levels.

#### 5.1.4 Analysis of the effects of educational level on nutritional literacy

The results indicate that educational level plays a significant role in shaping nutritional literacy among older adults within this study's model. According to the Knowledge-Attitude-Practice theoretical framework, an individual's knowledge level directly influences their behavioral outcomes (43), which supports the reliability of our findings. Another survey on the nutritional literacy of the elderly from Spain, although using different versions of the questionnaire (44), also confirmed the positive impact of educational level on the nutritional literacy of the elderly. Therefore, local governments and community workers should intensify nutrition-related education campaigns tailored to older adults' comprehension capacities (e.g., auditory, visual, and interactive formats), which could achieve breakthroughs in improving their nutritional literacy. Regarding the negative correlation between denture use and nutritional literacy observed in this study, a plausible explanation lies in the critical impact of oral health—particularly dental status—on food choices among older adults. Limited healthcare resources in rural China often prevent scientifically optimal dental treatments post-tooth loss, resulting in poorly fitted dentures that compromise chewing efficiency (45). Studies also suggest that full-denture wearers tend to prefer less nutritious food options (12), increasing malnutrition risks. This phenomenon warrants further exploration in future research.

### 5.2 Analysis of the mediating effect of self-efficacy on nutritional literacy among older adults based on the TPB model

In this study, elderly self-efficacy was selected as the implementation intention and validated using structural equation modeling. The results revealed that self-efficacy plays a partial mediating role in the relationships between nutritional attitudes affecting nutritional literacy, and daily living abilities affecting nutritional literacy. As a positive psychological resource, self-efficacy can be used to assess older adults' confidence in their ability to acquire nutrition when engaging in nutritional behaviors. With the development of social psychology, the impact of positive psychological resources on health-promoting behaviors has garnered widespread attention, and an increasing number of studies have focused on the relationship between psychological changes in the elderly and their health status (46, 47). Within this field, research on the relationship between self-efficacy and health-promoting behaviors among older adults has been extensively explored (48). This relationship refers to whether older adults can adopt adaptive behaviors to mitigate health risks when facing environmental stressors or challenges, with self-efficacy being a psychological cognition closely associated with such behaviors (49–51). Positive nutritional attitudes may enhance self-efficacy by fostering a growth mindset. For instance, elderly individuals who value balanced diets (attitude) are more likely to persist in overcoming dietary challenges (e.g., tooth loss), thereby strengthening confidence (self-efficacy). This aligns with Bandura's social cognitive theory, where mastery experiences (successful behavior attempts) build efficacy beliefs (52). Existing studies have confirmed that self-efficacy among rural elderly in China is significantly lower compared to their urban counterparts (53). Therefore, prioritizing the self-efficacy of rural elderly populations will be a critical measure to enhance their nutritional literacy.

## 6 Conclusions

In summary, our results extend the TPB by emphasizing self-efficacy as a critical mediator in resource-constrained environments. This suggests that interventions targeting rural older adults should combine attitude transformation with efficacy enhancement—for example, by increasing their motivation to obtain nutrition, strengthening multi-level social support (e.g., community assistance, family care), and improving daily living assistance for functionally impaired individuals. Simultaneously, addressing the psychological well being of rural older adults to elevate their self-efficacy will significantly improve their nutritional literacy. Both societal and familial strategies to enhance nutritional literacy in aging populations can reference the TPB framework. This “attitude-efficacy” dual intervention model may narrow the intention-behavior gap predicted by the TPB, offering a novel framework for global aging initiatives.

## 7 Limitations

This study represents the first investigation into the nutritional literacy of the elderly population in rural areas of northern Sichuan. The sample selection has limitations in quantity for the local rural elderly population. To further validate the research findings, future studies could expand the sample size to enhance the generalizability of conclusions.

While the selected villages (Sanlin, Puji, and Luhua) reflect typical socioeconomic and dietary characteristics of northern Sichuan's rural elderly population, the convenience sampling method limits the generalizability of findings to broader rural or international aging cohorts. Future studies should adopt stratified or cluster sampling across diverse regions (e.g., coastal vs. inland, varying economic tiers) to enhance representativeness. Additionally, cultural and infrastructural differences between rural China and other countries necessitate caution when extrapolating results globally.

Dietary habits and lifestyle form the foundation of nutritional literacy, and thus their nutritional literacy status may ultimately evolve in ways that influence chronic disease outcomes. As this study employed a cross-sectional survey design, it did not investigate the pre-morbid nutritional literacy or disease progression timelines of elderly individuals with chronic conditions. Therefore, more longitudinal research is needed to explore the impact of chronic diseases on nutritional literacy.

This study has limitations in measuring perceived behavioral control (PBC). While the IADL scale captured functional capacity (e.g., cooking, shopping), it overlooked resource accessibility (e.g., income, geographic proximity to markets) and cultural factors (e.g., traditional dietary preferences for preserved foods). Additionally, the cross-sectional design ignored dynamic resource fluctuations (e.g., seasonal income changes). Future research should integrate multidimensional PBC indicators (e.g., economic subsidies, cultural attitudes) and longitudinal assessments to address these gaps.

## Data Availability

The datasets presented in this study can be found in online repositories. The names of the repository/repositories and accession number(s) can be found here: doi: 10.6084/m9.figshare.28396298.
